# *Protium spruceanum* Extract Enhances Mupirocin Activity When Combined with Nanoemulsion-Based Hydrogel: A Multi-Target Strategy for Treating Skin and Soft Tissue Infections

**DOI:** 10.3390/pharmaceutics16060700

**Published:** 2024-05-23

**Authors:** Tatiane Roquete Amparo, Lucas Resende Dutra Sousa, Viviane Flores Xavier, Janaína Brandão Seibert, Débora Luiza Paiva, Débora dos Santos da Silva, Luiz Fernando de Medeiros Teixeira, Orlando David Henrique dos Santos, Paula Melo de Abreu Vieira, Gustavo Henrique Bianco de Souza, Geraldo Célio Brandão

**Affiliations:** 1 Department of Pharmacy, Federal University of Ouro Preto, Ouro Preto 35400-000, Brazil; lucasresendedutrasousa@gmail.com (L.R.D.S.); viviane.xavier@aluno.ufop.edu.br (V.F.X.); paiva.farm@gmail.com (D.L.P.); deborasilva.farma@gmail.com (D.d.S.d.S.); orlando@ufop.edu.br (O.D.H.d.S.); guhbs@ufop.edu.br (G.H.B.d.S.); celiobrandao@ufop.edu.br (G.C.B.); 2 Laboratory of Pathology and Microbial Control, University of São Paulo (USP-ESALQ), Piracicaba 13418-900, Brazil; janainaseibert@usp.br; 3 Department of Clinical Analyzes, Federal University of Ouro Preto, Ouro Preto 35400-000, Brazil; lfmt@ufop.edu.br; 4 Department of Biological Sciences, Federal University of Ouro Preto, Ouro Preto 35400-000, Brazil; paula@ufop.edu.br

**Keywords:** SSTIs, antibacterial, nanoemulsion-based hydrogel, *Protium spruceanum*, synergistic activity

## Abstract

The treatment of skin and soft tissue infections (SSTIs) can be challenging due to bacterial resistance, particularly from strains like MRSA and biofilm formation. However, combining conventional antibiotics with natural products shows promise in treating SSTIs. The objective of this study is to develop a nanoemulsion-based hydrogel containing *Protium spruceanum* extract and mupirocin and evaluate its potential for the treatment of SSTIs. The nanoemulsion was obtained by phase inversion and subsequently characterized. The antibacterial activity was evaluated in vitro against *S. aureus* MRSA, including the synergism of the combination, changes in membrane permeability using flow cytometry, and the anti-biofilm effect. In addition, the irritative potential was evaluated by the HET-CAM assay. The combination exhibited synergistic antibacterial activity against *S. aureus* and MRSA due to the extract enhancing membrane permeability. The hydrogel demonstrated suitable physicochemical properties, inhibited biofilm formation, and exhibited low irritation. The formulation was nanometric (176.0 ± 1.656 nm) and monodisperse (polydispersity index 0.286 ± 0.011). It exhibited a controlled release profile at 48 h and high encapsulation efficacy (94.29 ± 4.54% for quercitrin and 94.20 ± 5.44% for mupirocin). Therefore, these findings suggest that the hydrogel developed could be a safe and effective option for treating SSTIs.

## 1. Introduction

Skin and soft tissue infections (SSTIs), such as impetigo, scalded skin syndrome, erysipelas, cellulitis, folliculitis, carbuncles, and fasciitis, are among the most frequent bacterial infections and are associated with a significant increase in costs and the rate of hospitalizations, as a serious health problem [1,2,3]. One of the factors of concern is the prevalence of resistant strains, such as methicillin-resistant *Staphylococcus aureus* (MRSA), which is responsible for SSTIs worldwide [3,4].

The increase in infections by multidrug-resistant bacteria, mainly MRSA, complicates the treatment and intensifies morbidity and mortality numbers [5]. In addition to the occurrence of resistant strains, another factor that causes concern in relation to *S. aureus* infections is the ability to form biofilms [6,7]. Biofilms are the primary cause of impaired wound healing, resulting in the development of chronic wounds. They can also enhance the resistance of bacteria to antibiotics, making treatment challenging [7,8,9].

The primary treatment for most cases of SSTIs is through procedures such as drainage and incisions. However, antibiotics are also used that accelerate healing and reduce the rate of recurrence [10]. Mupirocin is one of the most effective drugs in the treatment of topical SSTIs by *S. aureus*. Its effectiveness is compared to other topical antimicrobials, such as neomycin, and also to oral antimicrobials, such as erythromycin [11]. However, the resistance of *S. aureus* strains to mupirocin reduces the effectiveness of this drug [10].

One of the strategies that can be used to improve the treatment of infections caused by multidrug-resistant strains is the combination of conventional antibiotics with natural products [12]. *Protium spruceanum* (Benth.) Engler, a Brazilian native species from the Burseraceae family, is popularly known as “breu” [13]. The hydromethanolic extract of the leaves has a bactericidal action, especially against *S. aureus* [14]. In addition to its antimicrobial action, this extract also has anti-inflammatory and antioxidant activities, which are associated with the presence of flavonoids [15]. Allied with antibacterial, these activities can accelerate the healing process, preventing complications with chronic infections [16,17].

In addition to its synergism and multi-target action, nanotechnology can also facilitate the treatment of SSTIs. Nanoemulsions can improve the anti-biofilm action and increase skin penetration [18,19]. However, the low viscosity of nanoemulsions can make topical application difficult, which justifies incorporation in hydrogel [20]. Thus, the objective of this present study is the development of a nanoemulsion-based hydrogel containing the hydromethanolic extract of *P. spruceanum* leaves and mupirocin, aiming at the treatment of SSTIs.

## 2. Materials and Methods

### 2.1. Plant Material and Extraction

The leaves of *P. spruceanum* were collected in February 2019 at Lavras, Minas Gerais, Brazil (coordinates 21°17′33.6″ S and 44°59′15.1″ W; 21°18′11.9″ S and 44°59′18.8″ W). A voucher for the species (16399 HESAL) was deposited in the Herbarium of Universidade Federal de Lavras. The hydromethanolic extract was obtained by sequential extraction and assisted by ultrasound using hexane and a methanol/water (7:3) mixture in a 40 kHz ultrasound bath (USC 1400, Unique, Indaiatuba, Brazil) at 40 °C, as previously reported [21]. The access for this research is registered at SisGen, Brazilian National System of Genetic Resource Management and Associated Traditional Knowledge under number AF47CE9.

### 2.2. Quercitrin and Mupirocin Quantification

The analysis was performed on a Waters Alliance HPLC-DAD system (Waters, Milford, MA, USA) equipped with a C-18 column (Luna, 4.6 mm × 250 mm, 5 μm particle size) (Phenomenex, Torrance, CA, USA) at 30 °C. The mobile phase comprised 0.5% acetic acid in Milli-Q purified water (solvent A) and methanol (solvent B). The flow rate was 1.0 mL/min, and the injected volume was 20 µL, using the gradient (10–20% B/0–5 min, 20–40% B/5–10 min, 40–50% B/10–15 min, 50–60% B/15–20 min, 60–100% B/20–30 min, 100–10% B/30–35 min, 100% B/35–40 min).

Quercitrin, a major flavonoid already isolated and identified from *P. spruceanum* leaves [22], was used as a marker for extract quantification. Quercitrin and mupirocin standard solutions (500 μg/mL in methanol) were used to obtain the range of concentration of 5–250 μg/mL. In total, a 254 nm wavelength was used for quercitrin analysis and 230 nm for mupirocin. The analytical method was semi-validated to demonstrate selectivity, linearity, precision (repeatability), and accuracy [23,24].

### 2.3. Development and Characterization of Hydrogel

#### 2.3.1. Preparation of Nanoemulsion-Based Hydrogel

The nanoemulsion was obtained by the phase inversion emulsification method (PIE), as previously reported, with modifications [25]. The hydrogel was obtained by the addition of a carbopol solution to the nanoelmulsion at a ratio of 1:1 under magnetic stirring. The final composition of the nanoemulsion-based hydrogel was as follows: Sorbitan oleate (Span 80—Croda, Campinas, Brazil) (1.50% *w*/*v*), Polysorbate 80 (Tween 80—Synth, São Paulo, Brazil) (3.50% *w*/*v*), corn oil (2.50% *w*/*v*), DMSO (0.5% *w*/*v*), *P. spruceanum* extract (0.25% *w*/*v*), mupirocin (0.025% *w*/*v*), Carbopol ultrez 20 (Lubrizol, Wickliffe, OH, USA) (0.60%) and distilled water (83.45% *w*/*v*). 

#### 2.3.2. Hydrogel Characterization

The particle size and polydispersity index (PDI) were measured by photon correlation spectroscopy and zeta potential by electrophoretic mobility through Zetasizer (Malvern, model Zetasizer Nano series—Nano ZS; Malvern Panalytical, Westborough, MA, USA). 

The measurements of particle mean size, PDI, zeta potential, and pH were conducted for all samples immediately after processing and again at intervals of 1, 7, 14, and 28 days to study the stability of samples maintained at room temperature.

Rheological parameters of the gel were determined using a Brookfield DV-III rheometer at 25 °C, spindle S2 (CPES2), with 0.5 g of the sample. Measurements were made using a rotational speed of 2–12 rpm, varying in intervals of 2 rpm every 30 s to obtain an upward curve. The downward curve was obtained by decreasing this rotation with the same intervals. Flow and viscosity curves were obtained by determining the shear rate as a function of the tension (flow curve/rheogram) and viscosity (viscosity curve). The results were adjusted to the Bingham, Casson, Herschel–Bulkley, and Ostwald–de-Waele (Power Law) models [26].

Encapsulation efficiency (EE) was determined using ultracentrifugation/ultrafiltration in microtubes assay, and the drug release study was performed using a dialysis membrane with ethanol at 50% *v*/*v* in PBS and pH 7.2 as the receptor medium [27]. The in vitro drug release study was performed using the dialysis membrane (SnakeSkinTM Dialysis Tubing). A total of 200 μL of hydrogel was added to the donor compartment, and it was placed in contact with the receptor medium under stirring at 37 °C (Fisher Scientific, Waltham, MA, USA) [28]. Phosphate buffer solution (PBS) with tween 80 (10%) was used as the receptor medium (pH 7.2). Aliquots were removed from the receptor medium at specific intervals (1, 2, 3, 4, 5, 6, 8, 10, 12, 24, and 48 h), and the same volume of fresh solutions was replaced to maintain the sink conditions. Extract and mupirocin were quantified by the validated HPLC method previously described. 

Mupirocin, the extract, and hydrogel with and without actives were analyzed by Fourier Transform Infrared (FTIR) Spectroscopy analysis using a Shimadzu IRPrestige-21 FTIR spectrometer (Shimadzu, Kyoto, Japan) with a background run used as a negative control. The spectra used ranged from 400 to 4000 cm^−1^ with a resolution of 4 cm^−1^ and 32 scans [29].

### 2.4. Antibacterial Activity

The in vitro antibacterial activity was evaluated against *Staphylococcus aureus* ATCC 25923 and *Staphylococcus aureus* MRSA ATCC 43300 cultivated in Müeller–Hinton agar (MHA) medium at 37 °C for 24 h before each assay. The inoculums were prepared using the direct colony suspension method by means of the saline (0.9 % NaCl) suspension of colonies selected from a 24 h agar plate before each assay. The suspension was adjusted to reach turbidity equivalent to 0.5 of the McFarland standard scale (1 × 10^8^ CFU mL^−1^) [30].

### 2.5. Evaluation of the Synergistic Potential

Aiming to evaluate the synergism of the extract combined with mupirocin, the minimum inhibitory concentration (MIC) of this drug (2.0 to 0.015 µg/mL), extract (8000 to 60 µg/mL), and combinations were determined through the broth microdilution method [30]. The 96-well plates were incubated at 37 °C for 24 h. After the incubation period, 30 µL of triphenyl tetrazolium chloride (TTC, 0.25 mg/mL) was added, and the plates were incubated for an additional 3 h, and the MIC was determined as the smallest concentration without visible color. 

The Fractional Inhibitory Concentration Index (FICI) was calculated according to Goto, Hiramatsu, and Nasu [31] as follows: FICI = (MIC of the extract in combination/MIC of the extract) + (MIC of mupirocin in combination/MIC of mupirocin). The results were classified as synergism (FICI < 1), indifferent (1 < FICI < 2), and antagonism (FICI > 2) [32].

### 2.6. Cytoplasmic Membrane Permeability

Flow cytometry, using propidium iodide (PI) as a marker, was used to evaluate cell membrane permeability changes [25]. The inoculums were diluted 1:100 in Müeller–Hinton broth (1 × 10^6^ CFU/mL). This procedure was followed by the addition of 100 μL of treatments consisting of 2× MIC of mupirocin, the extract, combination or broth only (negative control), and 100 μL of inoculums for each well in a 96-well plate, incubated for 24 h at 37 °C. After treatment, the samples were transferred to cytometry tubes, centrifuged, and washed with phosphate-buffered saline. Cells were resuspended in 100 μL of the same buffer and incubated with 1 μL of the PI solution (1 mg/mL) for 30 min at 4 °C protected from light. The dialing control was prepared in the same way as the negative controls but without a PI solution. Flow cytometry analyses were conducted using BD FACS Calibur (Becton Dickinson Bioscience, San Diego, CA, USA), and PI was excited by the blue laser (438 nm) and analyzed by the FL3 channel 670 LP). The data were analyzed using the software Flow Jo v.10.

### 2.7. Anti-Biofilm Effect

The inoculum was adjusted in broth in order to obtain 1 × 10^7^ CFU/mL. The hydrogel was diluted in Müeller–Hinton broth (final concentrations 0.5× MIC, MIC, and 2× MIC) and added in 96-well tissue culture microtiter plates along with the inoculum. After incubation for 24 h/37 °C, the medium was aspirated, and the wells were washed with saline to remove unattached bacteria and dried at 50 °C for 40 min. Then, the biofilms were fixed by adding methanol (200 μL) for 15 min and dried again. The wells were stained with 0.1% crystal violet (200 μL) for 5 min. The excess stain was rinsed off with water, and after drying the stain, it was resolubilized in 100 μL of ethanol. The absorbance was measured at 570 nm, and the biofilm percentage was determined in relation to the control (without treatment) [33]. 

In addition, microscopy images were obtained to visualize the biofilm formed. After treatment, the bacteria were fixed with 3% glutaraldehyde in a 0.1 M phosphate buffer, pH 7.3, serially dehydrated in ethanol (70%, 95%, and 100%, 2 min in each) and dried again at 50 °C. Gram staining was used for the optical microscope, 4’,6-diamidino-2-phenylindole (DAPI, 2 µg/mL) to stain intracellular DNA in bacteria, which was used for fluorescent microscopy and a small amount of carbon was sputtered on the samples for scanning electronic microscopy (SEM).

### 2.8. HET-CAM Test

The chorioallantoid membrane of the fertilized chicken eggs test (HET-CAM) was performed for the evaluation of the hydrogel irritative potential [34]. The procedure obtained commercially fertile White Leghorn chicken eggs (Granja Tolomei, Rio de Janeiro, Brazil) without mycoplasms, and these were used after the 10th day of incubation at 38 °C. The eggs were opened near the air cell using surgical scissors to reveal the highly vascularized chorioallantoic membrane (CAM). After 20 s of the product application (0.1 mg), the CAM surface was rinsed with saline solution and observed over a period of 300 s, and the time at which the appearance of hemorrhage, lysis, and coagulation occurred was recorded. The positive irritant control was 0.1 mol/L sodium hydroxide, and the negative control was 0.9% sodium chloride. For each sample, 3 eggs were used.

The irritant effects were classified by scores according to the time they were observed: less than 30 s (hyperemia: 5; hemorrhage: 7; clot formation/opacity: 9); between 30 and 120 s (hyperemia: 3; hemorrhage: 5; clot formation/opacity: 7); or between 120 and 300 s (hyperemia: 1; hemorrhage: 3; clot formation/opacity: 5). If an effect was not observed after 300 s, it was scored as zero. The hydrogel was classified according to the mean value scores of three eggs: 0–4.99 corresponding to non-irritant/slightly irritant (NI/SI); 5.00–8.99 corresponding to moderately irritant (MI); and 9.00–21.00 corresponding to severely irritant (SVI).

### 2.9. Statistical Analysis

The normal distribution of the presented data was verified using the Shapiro–Wilk test. The results are presented as the mean and standard error and were analyzed by an analysis of variance (ANOVA) followed by a comparison with Dunnett’s post-test using Graph Pad Prism 5.0 software. Differences between days 1, 7, 14, and 28 after the development of nanoemulsion were analyzed using the *t* test for Zeta potential, hydrodynamic size, and the polydispersity index using Graph Pad Prism 8.0.1. The significance level was *p* < 0.05. 

## 3. Results and Discussion

### 3.1. Antibacterial Activity Synergic

The increasing prevalence of staphylococcal resistance to conventional antibiotics in SSTIs is a global concern [35]. Therefore, researching the current antibacterial effect of antibiotics, such as mupirocin, in association with natural compounds is important to facilitate the treatment of infections caused by multi-resistant bacteria [12]. The activity of the hydromethanolic extract of *P. spruceanum* against *S. aureus* has already been reported by Amparo et al. [36], but its effect, in combination with other antibiotics such as mupirocin, has not yet been studied. 

The results showed that ICIF values for *S. aureus* and MRSA were below 1 (Table 1), indicating a synergistic effect between the *P. spruceanum* extract and mupirocin for these two bacterial strains [21].

An increasing number of studies are investigating the synergistic effects of antibiotics and plant extracts as a potential treatment for infections. One found a synergistic effect between mupirocin and isoflavonone bidwillon B against MRSA strains [37]. Another study evaluated the synergistic effect of propolis, which contains flavonoids among other compounds, with mupirocin. They found that the combination of mupirocin and propolis reduced bacteria in the nostrils of rabbits more effectively than treating each one separately [38]. In this sense, these results support our work and suggest that using our samples together may be beneficial in fighting multi-resistant bacteria.

The synergism between the extract and mupirocin may be attributed to the increase in membrane permeability resulting from the extract treatment. This result was observed in the flow cytometry using the propidium iodide (PI) assay. When PI passes through the membrane and binds to DNA, it emits fluorescence, which can be quantified using the flow cytometry technique [39]. PI penetration is directly related to the loss of structural integrity and, consequently, to the impairment of the transport function across the plasma membrane [40,41]. 

**Figure 1:**
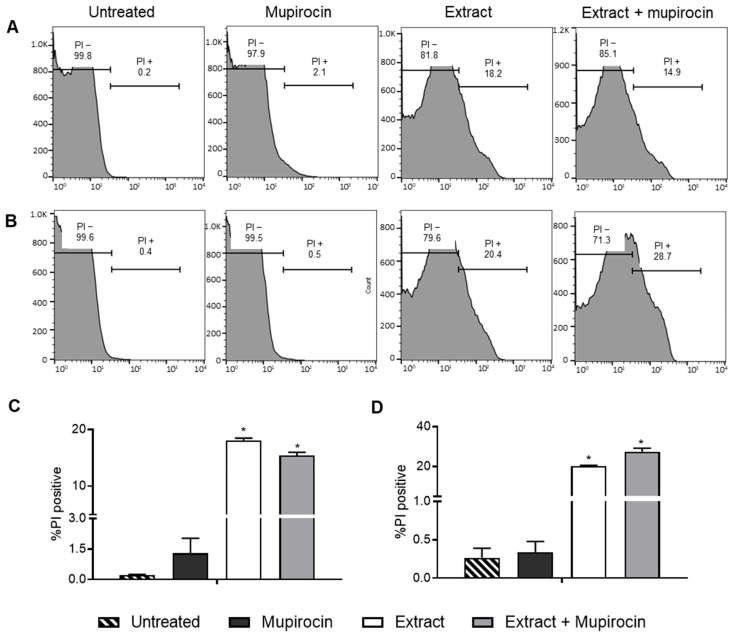
Flow cytometry analysis of *Staphylococcus aureus* (**A**,**C**) and MRSA (**B**,**D**) membrane permeability for the control (untreated cells) and after treatment with *Protium spruceanum* extract and/or mupirocin. (**A**,**B**) Histograms; (**C**,**D**) Percentage of damaged cells stained by propidium iodide (PI). (*) denotes a significant difference compared to the untreated cells (*p* ≤ 0.05), as determined by one-way ANOVA followed by Dunnett’s post-test.

It is worth noting that treatment with the extract associated with the antibiotic induced an increase in membrane permeability for both bacteria. The isolated extract samples also exhibited an increase in permeability in relation to the two bacteria, but this effect was not observed for the antibiotic alone (Figure 1). In the literature, it is possible to find other studies that address the effect of natural products on the bacterial membrane. In the study carried out by Weng et al. [42], for example, the authors found that the flavonoids oforaflavanone G and kurarinone have significant antibacterial activity and anti-resistance properties. Such properties were attributed, among other factors, to the destruction of membrane integrity.

The composition of the *P. spruceanum* fraction includes flavonoids, among other compounds [22]. Other studies have shown that the presence of flavonoids in combination can lead to an increase in membrane permeability. Eumkeb et al.’s [43] study demonstrated that the combination of flavones with amoxicillin increased the membrane permeability of microorganisms, whereas the two alone did not exhibit this effect. Another study showed that the antibacterial activity of the flavonoids catechin, epicatechin gallate, and 3-O-octanoyl is associated with moderate and high membrane permeability [44]. This supports the theory that the flavonoids in the *P. spruceanum* composition are responsible for the synergistic effect on membrane permeability. 

**Figure 2:**
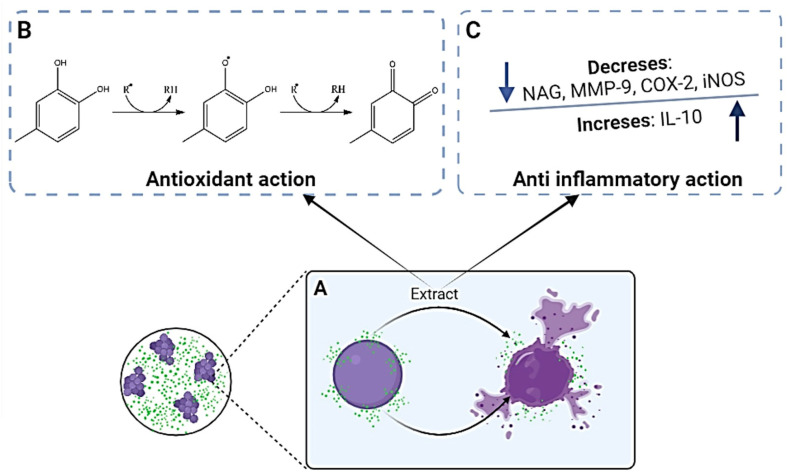
Schematic representation of the mechanism of antibacterial (**A**), antioxidant (**B**), and anti-inflammatory (**C**) actions of the hydromethanolic extract of *Protium spruceanum*.

One of the mechanisms by which bacteria develop resistance is through the reduction in membrane permeability, resulting in the decreased absorption of antibiotics [45]. The fraction associated with mupirocin demonstrated an increase in membrane permeability. This increase can potentially reduce bacterial resistance and enhance the effectiveness of the antibiotic by facilitating its entry into the bacterial cell (Figure 2A).

In addition to the advantage of better antibacterial effects, combining mupirocin with the *P. spruceanum* extract also has other advantages. This extract has antioxidant action related to its ability to scavenge free radicals (Figure 2B) and anti-inflammatory activity due to an immunomodulatory effect (Figure 2C), allowing a multi-target outcome that may improve the treatment of SSTIs [36]. Thus, the combination of the *P. spruceanum* extract and mupirocin holds promise for treating SSTIs and was utilized in the development of a nanoemulsion-based hydrogel.

### 3.2. Nanoemulsion-Based Hydrogel Development and Determination of Nanoparticle Size and Zeta Potential

Topical administration allows the delivery of the drug directly to the site of infection. Therefore, in relation to systemic treatment, the main advantages are as follows: increasing bioavailability at the site of infection, combating resistant microorganisms, minimizing systemic toxicity and adverse effects, increasing patient therapeutic adherence, and reducing treatment costs [46].

Resistance to systemic antimicrobials leads to the need to use drugs in higher and more toxic doses. Therefore, the treatment of SSTIs with topical antimicrobials is a good alternative [46]. In this sense, a nanoemulsion-based hydrogel containing the hydromethanolic extract of *P. spruceanum* leaves and mupirocin was developed aimed at the topical treatment of SSTIs.

It is essential to evaluate the physical–chemical characteristics of the developed systems, such as their size, polydispersity index, and surface charge (Zeta potential). These parameters provide information regarding the permeation capacity of the active ingredients in question in the skin and formulation stability [47,48,49,50].

The developed formulation was found to be nanometric and monodisperse, with a size of 176.0 ± 1.656 nm and a polydispersity index of 0.286 ± 0.011 [47,48,49,50]. Compared to the formulation without actives (size 125.9 ± 1.50 nm and PI 0.204 ± 0.02), the addition of extract and mupirocin exhibited a larger size, indicating the encapsulation of the compounds. 

**Figure 3:**
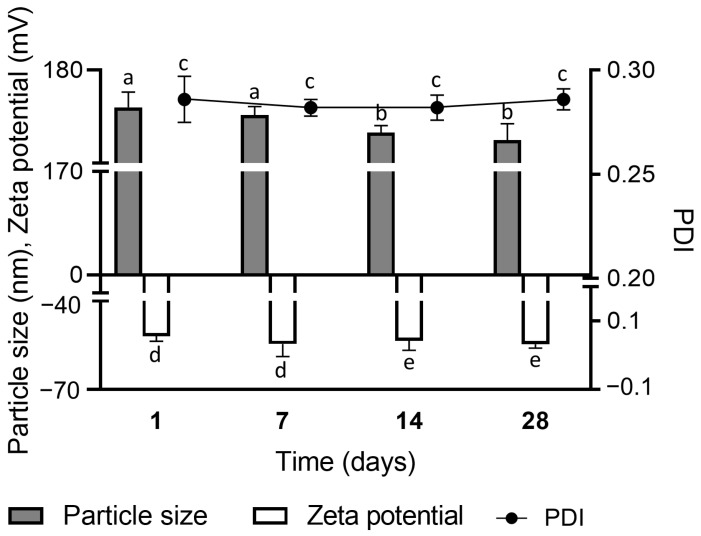
Stability of the nanoemulsion-based hydrogel containing *Protium spruceanum* extract and mupirocin stored at room temperature for 28 days. Different lowercase letters indicate a significant statistical difference between the same sample in different periods (*p* < 0.05), as determined by the *t* test.

The zeta potential value of the nanoemulsion-based hydrogel was −51.9 ± 1.79 mV. This result indicates the strong tendency of the formulation to remain stable since zeta potential zeta greater than 30 mV in the modulus is considered adequate for an electrostatic repulsion between the particles, consequently preventing their aggregation, decantation, and instability over time [25]. This indicator of stability is supported by the narrow difference in particle size, polydispersity index (PDI), and zeta potential in the hydrogel stored at room temperature for 28 days (Figure 3).

In terms of the topical application of the developed nanoemulsion-based hydrogel, it is believed that the combination of mupirocin and the *P. spruceanum* extract would be effective due to the small droplet sizes, which could enhance their skin penetration and subsequent action [51].

### 3.3. Rheological Behavior of the Nanoemulsion-Based Hydrogel

The results of the rheological analyses were well-adjusted to the Herschel–Bulkley model with r = 0.995 (r > 0.99), which considers the non-linear relationship between the stress and the shear rate (non-Newtonian behavior) [26]. The parameters of the Herschel–Bulkley equation adjusted to the flow curves indicated a flow index (n) of 0.65 ± 0.09, consistency index (k) of 6153 ± 162, and stress index of 91.9 ± 12.4. 

**Figure 4:**
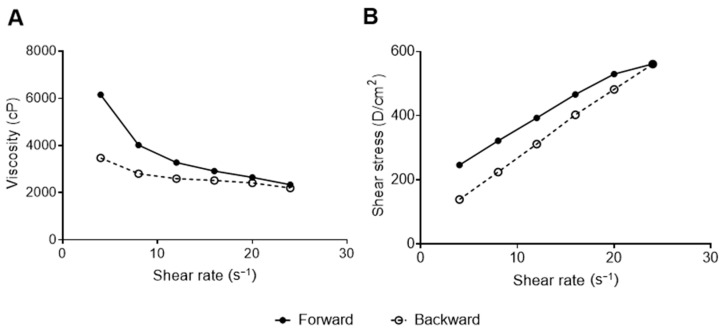
Steady shear flow curve (**A**) and rheogram (**B**) of the nanoemulsion-based hydrogel containing *Protium spruceanum* extract and mupirocin.

The result n < 1 confirms that the developed gel is classified as non-Newtonian fluid and indicates that it should be considered as pseudoplastic behavior [52,53,54]. The steady shear flow curve (Figure 4A) shows this pseudoplastic behavior in which the viscosity decreases with the increase in the shear rate [52,53,54]. 

Pseudoplasticity can facilitate the clinical administration of semi-solid systems for topical application because, at high shear rates (expulsion from the recipient) the material flows readily, and at low shear rates (spread gel) the material adopts a higher consistency, recovering the original rheological properties before administration [53,54]. 

The rheogram (Figure 4B) indicates thixotropic behavior, in which the forward and backward curves do not coincide and form a hysteresis loop [53,54]. The thixotropic behavior of gels is a desirable propriety that allows its initially thick application on the skin of a product, which, under shear stress, becomes thinner and easily spreadable material [55].

### 3.4. Validation of the Method for Quercitrin and Mupirocin Quantification

The purpose of validating the analytical method is to attest to the efficiency of reproducing valid analytical results [56]. To evaluate encapsulation efficiency and release, we validated a quantification method in LC-DAD. Quercitrin was selected as an extract marker due to its prevalence as the major component in the hydromethanolic extract of *P. spruceanum* leaves [15].The analytical method was subjected to the semi-validation of chromatographic parameters in accordance with current Brazilian legislation (RDC 166/2017) [24], and the results are presented in Table 2.

The objective of evaluating the selectivity of the method is to verify whether it can distinguish the response of analyte interest in the presence of other analytes [24,57]. It is important to highlight that the developed method produces responses to other analytes present in the nanoemulsion-based hydrogel [15]. However, the results obtained show that the method is selective and presents good resolution between peaks and peak purity. The purity angle was lower than the purity threshold, confirming the selectivity of the method.

Validation results for the quantification method of quercitrin and mupirocin showed that there is a high linear correlation for the validated method in the range of 5 to 250 µg/mL. The angular coefficients are significantly different from zero, and there was no significant deviation from linearity. According to Moretto and Calixto [57], when the correlation coefficient is greater than 0.999, it can be considered that there is an ideal relationship between the data. The closer this value is one, the smaller the dispersion of results around the line of best fit. In this way, it can be stated that area and concentration values are highly correlated in this concentration range; any increase or decrease in concentration causes, proportionally, an increase or reduction in the response [24].

The method was precise and accurate since it showed an RSD value smaller than 5%. The results obtained for this method in the precision test showed little dispersion around the average curve; that is, the experimental points tended to be concentrated close to the average curve. According to Taveniers et al. [58], for the analytical method to be considered accurate, DPR values must be less than 5%. The results obtained show that the method is capable of providing consistent and accurate results, even when repeated several times. The accuracy of the developed analytical method was verified using three concentrations (minimum, average, and maximum) that covered the linear range and was proven by demonstrating the degree of agreement between individual results in relation to the average value accepted as true [24].

Therefore, the validated method was considered suitable for analyzing the encapsulation efficiency and release profile of the nanoemulsion-based hydrogel containing *P. spruceanum* extract and mupirocin.

### 3.5. Encapsulation Efficiency and Release

The encapsulation efficiency was similar for the two active ingredients evaluated, which were 94.29 ± 4.54% for quercitrin and 94.20 ± 5.44% for mupirocin. These are factors that determine the efficiency of encapsulation, such as the nature of the encapsulated material, total solids content, and preparation method used to obtain the particles [59,60]. Given this, it can be stated that in this study, the choice of the homogenization method and the surfactants used were appropriate and allowed particles to be obtained with high encapsulation efficiency.

**Figure 5:**
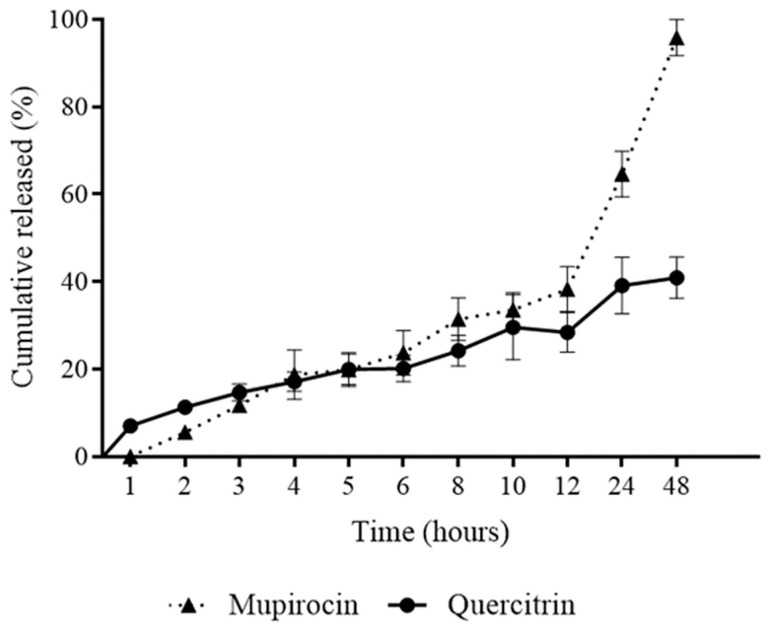
Release profile of nanoemulsion-based hydrogel containing *Protium spruceanum* extract and mupirocin.

In relation to the release profile of the hydrogel, the results were expressed by relating the amount of accumulated mass of quercitrin and mupirocin released (%) as a function of the collection time in Figure 5.

The initial liberation profile shows that the nanoemulsion-based hydrogel released approximately 30% of quercitrin in the first 12 h of evaluation and, from that moment on, the nanoemulsion-based hydrogel exerted greater release control, so that release was sustained until the end of the experiment when there was a liberation approximately 40% of quercitrin. Regarding the liberation of mupirocin, this presented a release profile similar to quercitrin in the first 12 h; however, from that moment onwards, it was possible to observe the increasing release of mupirocin in relation to time until 48 h, when there was a release of approximately 95% of mupirocin.

In general, it can be seen that the nanoemulsion-based hydrogel presented a slow liberation profile. Despite having liberated only 40% of quercitrin, it is important to highlight that it was used as a phytomarker, as *P. spruceanum* presents other important active substances, such as quercetin and rutin, as demonstrated in the study by Amparo et al. [15], where, due to their presence in significant quantities in *P. spruceanum*, they were probably also released. Therefore, the liberation profile of the nanoemulsion-based hydrogel is suitable for the proposed use.

### 3.6. Fourier Transforms Infrared Spectrometry (FTIR)

**Figure 6:**
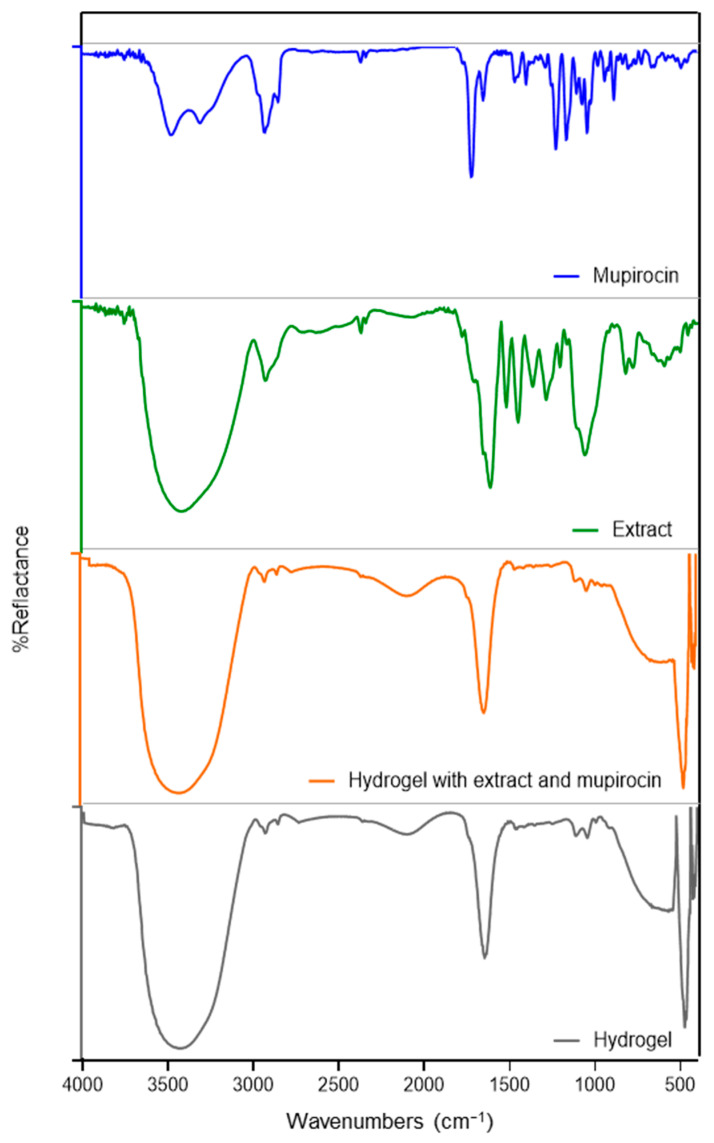
FTIR spectra for mupirocin, extract and nanoemulsion-based hydrogel with and without mupirocin and extract.

Figure 6 presents the FTIR spectra of the actives and hydrogels. Mupirocin exhibited bands at 3477.7 and 3309.9 cm^−^^1^, corresponding to O−H stretching, as well as dual bands at 2931.8 and 2852 cm^−^^1^, related to C–H stretching. The 1722.4 cm^−1^ band is associated with C=O strong stretching, while the 1166.9 cm^−1^ band is attributed to C–O strong stretching. The spectra are consistent with the chemical structure of this antibiotic and corroborate the findings of previous studies [29,61].

The extract under investigation was observed to exhibit the characteristic absorption bands of phenolic compounds, including flavonoids, which have already been reported to be the main constituents of this extract [14]. The band at 3415.9 cm^−1^ is related to O–H stretching, while the band at 2926.0 cm^−1^ is related to C–H stretching. The band at 1610.6 cm^−1^ may be attributed to the stretching vibration of C=C groups, which are indicative of aromatic ring deformations. The band at 1448.5 cm^−1^ is associated with the vibration of C–H and the stretching vibration of aromatics. The band at 1284.59 cm^−1^ could be related to the C–O stretching of phenols, while the bands at 819.7 and 775.4 cm^−1^ are associated with the out-of-plane C–H aromatic vibrations [62,63].

The hydrogels had a strong broad band at 3417.9 cm^−^^1^ corresponding to O-H stretching, which occurred as a result of intermolecular hydrogen bonding [29]. The other bands observed were related to other excipients present in the formulation, including carbopol, surfactants, and oil [61]. The spectra of the hydrogel, with and without the extract and mupirocin, were largely analogous (Figure 6), suggesting an overlap between the bands of active ingredients and excipients. This observation can be interpreted as confirmation of the successful entrapment of the extract and mupirocin without evidence of a significant chemical interaction between active ingredients and other formulation components [29]. This absence of chemical interactions observed by comparative FTIR between mupirocin and the flavonoids (the principal compounds of the extract) and the used excipients, such as carbopol and polysorbate 80, has previously been documented in other studies involving distinct formulations [29].

### 3.7. Antibacterial Activity of the Nanoemulsion-Based Hydrogel 

In this study, it was verified that the nanoemulsion-based hydrogel was able to maintain the antibacterial activity of the encapsulated active ingredients, with MIC corresponding to 0.6 and 0.06 µg/mL, equivalent to the extract and mupirocin, respectively, with both *S. aureus* and MRSA strains assayed. 

**Figure 7:**
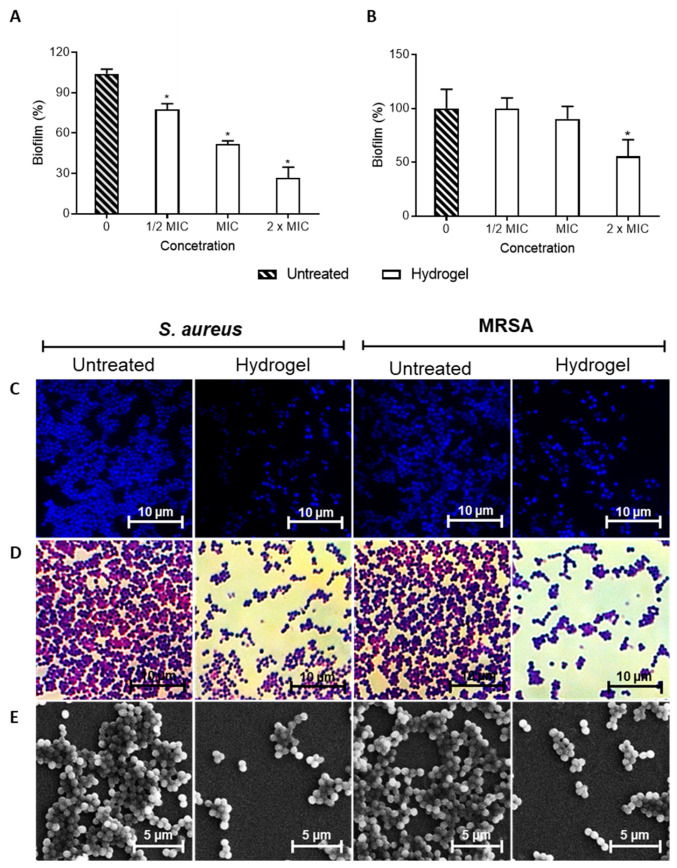
Efficiency of the nanoemulsion-based hydrogel containing *Protium spruceanum* extract and mupirocin in inhibiting biofilm formation. Percentage of biofilm inhibition related to untreated *Staphylococcus aureus* (**A**) and MRSA (**B**). Microscopy images with DAPI (**C**), Gram (**D**) stains, and scanning electron microscope (**E**). (*) denotes a significant difference compared to the untreated cells (*p* ≤ 0.05), as determined by one-way ANOVA, followed by Dunnett’s post-test.

In addition to determining the MIC, we evaluated whether the hydrogel could prevent the formation of biofilms, which are structures that can increase bacterial resistance and make SSTIs more difficult to treat [7,8,9]. The results obtained for the inhibition of biofilm formation are shown in Figure 7. It can be observed that the nanoemulsion-based hydrogel significantly reduced biofilm formation by *S. aureus*; this effect is directly proportional to the concentration tested. However, only doubling the MIC significantly decreased biofilm formation by MRSA.

Studies on the efficiency of new drugs in inhibiting biofilm formation are of great importance. According to Di Domenico and colleagues [64], 94.6% of *S. aureus* isolates with the MRSA phenotype are moderate-to-high biofilm-forming. *S. aureus* cells associated with the biofilm present differences in the cellular structure and are more resistant to antibiotics [65]. In general, nanoparticles have been widely used either alone or in combination with other antibacterial drugs against *S. aureus* biofilms [66,67]. 

In this study, the nanoparticles present in the nanoemulsion-based hydrogel were composed of *P. spruceanum,* which, among other compounds, presented phenolic compounds, including flavonoids, without its composition, as demonstrated by Amparo et al. [14]. Several phenolic compounds have already been shown to be effective against S. aueus biofilms, such as flavonoids, xanthohumol, gallic acid, proanthocyanidins, ellagic acid, tannic acid, ginkgolic acid and rosmarinic acid [68,69,70]. Therefore, the biofilm formation inhibitory activity (Figure 7) presented by the nanoemulsion-based hydrogel can be attributed to the presence of phenolic compounds in the composition of the extract.

Therefore, the nanoemulsion-based hydrogel developed is a promising option that has great application in the treatment of SSTIs.

### 3.8. HET-CAM Assay

The HET-CAM methodology is widely used and goes beyond research laboratories; this test is already used in the industry to identify potential non-irritating or moderately irritating materials during internal screening and safety assessments of formulations and/or raw materials of different physicochemical properties [71,72].

**Figure 8:**
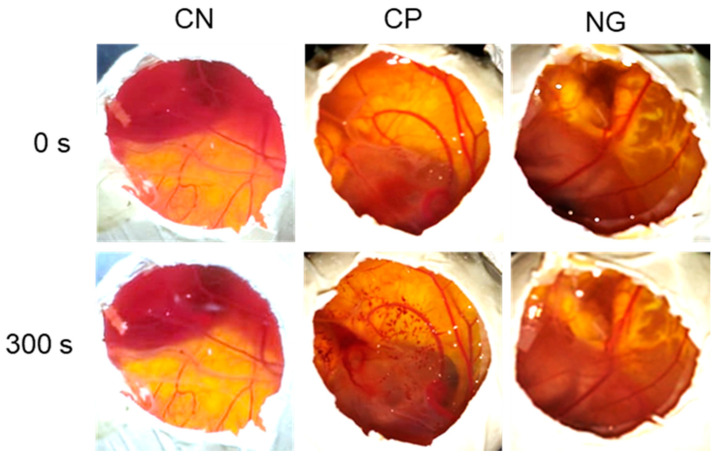
Macroscopic evaluation of the irritating potential of nanoemulsion-based hydrogel using the HET-CAM methodology. CN: Negative control; CP: positive Control; NG: nanoemulsion-based hydrogel.

The nanoemulsion-based hydrogel caused hyperemia, which was only observed after 300 s and was classified as low-irritating power, as shown in Table 3 and Figure 8. Furthermore, the results obtained demonstrate that the use of NaCl 0.9% and NaOH 0.1 mol/L controls was adequate. NaCl 0.9% was used as a negative control, and no aspects related to toxicity were observed. NaOH 0.1 mol/L was used as a positive control at the time of 30 s, and the occurrence of hyperemia and hemorrhage was observed; from 120 s onwards, in addition to hyperemia, the occurrence of coagulation was observed.

It is important to highlight that the HET-CAM test is accepted by regulatory agencies in European countries, such as Germany, France, and the United Kingdom. Furthermore, it was evaluated by experts who are members of the Interagency Coordination Committee on Validation of Alternative Methods (ICCVAM) and Interagency of the National Toxicology Program, Center for the Assessment of Alternative Methods of Toxicology (NICEATM), who published a protocol recommending the same for different lines of research [73]. This is an accepted and validated method in Brazil and is in accordance with CONCEA regulations Nº 18/2014 and Nº 31/2016 [74,75].

Considering the results of the low-irritating power of the nanoemulsion-based hydrogel containing *P. spruceanum* extract and mupirocin, it can be considered safe for topical use, and it can be recommended for other pre-clinical and clinical studies.

## 4. Conclusions

The hydromethanolic extract of *P. spruceanum* leaves exhibits synergistic antibacterial activity with antibiotic mupirocin against *S. aureus* and MRSA due to the extract’s ability to increase membrane permeability, which facilitates the entry of mupirocin into the cells. The hydrogel developed showed appropriate physicochemical parameters. Furthermore, it has the ability to inhibit biofilm formation and low irritation potential, indicating safety and efficacy. Therefore, these findings suggest that the developed hydrogel could be a promising option for treating SSTIs. 

## Figures and Tables

**Table 1:** Synergistic effect of *Protium spruceanum* extract and mupirocin antibiotic.

Bacteria	Extract	Antibiotic	Combination	ICIF
	MIC (μg/mL)	MIC (μg/mL)	MIC (μg/mL)	
*S. aureus*	2000 ± 577 ^a^	0.13 ± 0.07 ^a^	250 ± 72 *^a^/0.06 ± 0 ^#a^	0.62 ± 0.25 ^a^
MRSA	2000 ± 0 ^a^	0.06 ± 0.02 ^a^	125 ± 36 *^a^/0.03 ± 0.01 ^#a^	0.56 ± 0.17 ^a^

MIC: Minimum inhibitory concentration; ICIF: inhibitory concentration index fractionation; * = extract concentration; ^#^ = mupirocin concentration. Same lowercase letter indicate no significant statistical difference in the same column (*p* < 0.05).

**Table 2:** Parameters evaluated in the semi-validation of the analytical method.

Validation Parameter		Quercitrin	Mupirocin
Selectivity	Purity angle	1.267	0.726
	Purity angle	2.284	1.729
Linearity	r^2^	0.9999	0.9995
	Calibration curve	y = 31,807x − 2115	y = 9428x + 56,707
	Significance (a ≠ 0)	*p* = <0.0001 (a ≠ 0)	*p* = <0.0001 (a ≠ 0)
	Linearity deviation	*p* = 1.0000 (linear)	*p* = 1.0000 (linear)
**Parameter**	**Concentration (µg/mL)**	**RSD**	**RSD**
Intra-day precision	5	4.94	4.25
	10	4.88	4.60
	50	1.40	2.77
	100	1.37	4.89
	250	1.45	0.86
**Parameter**	**Concentration (µg/mL)**	**RSD**	**RSD**
Accuracy	5	4.13	4.25
	50	1.40	2.77
	250	1.45	0.86

**Table 3:** Results obtained in the evaluation of the irritating potential of the nanoemulsion-based hydrogel using the HET-CAM methodology.

Sample	30 s	120 s	300 s	Score
NaCl 0.9%	-	-	-	0
NaOH 0.1 mol/L	Hyperemia/hemorrhage	Coagulation/hyperemia	Coagulation/hyperemia	13.5
Nanoemulsion-based hydrogel	-	-	Hyperemia	0.5

## Data Availability

Data are contained within the article.
